# Supplementary material for: Very Low-Density Lipoproteins of Metabolic Syndrome Modulates STIM1, Suppresses Store-Operated Calcium Entry, and Deranges Myofilament Proteins in Atrial Myocytes
Source: J Clin Med. 2019 Jun 20;8(6):881. doi: 10.3390/jcm8060881 (PMC6617489; doi:10.3390/jcm8060881)
Supplement: Supplementary file 1 [file jcm-08-00881-s001.pdf]

Supplementary Table 1. The body and heart weights, echocardiographic measurements and biochemistry data in Normal-VLDL and MetS-VLDL injected mice.

	**Control (n= 4)**	**Normal-VLDL (n= 4)**	**MetS-VLDL (n= 4)**	**P value (ANOVA)**
Body weight (g)	32.3 ± 1.8	33.2 ± 1.9	35.1 ± 3.9	0.2903
Heart weight (mg)	216.0 ± 37.8	187.4 ± 106.9	304.0 ± 39.1[#]	0.0510
**Echocardiography Measurements**				
Left atrial diameter (mm)	2.14 ± 0.15	2.345 ± 0.40	2.82 ± 0.35[#]	0.0444
Left ventricular internal dimension (mm)	3.92 ± 0.11	3.90 ± 0.17	4.50 ± 0.30[#$]	< 0.001
Left ventricular wall thickness (mm)	0.94 ± 0.05	0.97 ± 0.20	0.87 ± 0.17	0.5646
LV mass (mg)	151.7 ± 12.5	150.8 ± 29.2	165.2 ± 37.3	0.6730
LV volume (μL)	66.8 ± 4.4	66.0 ± 6.9	93.0 ± 15.3[#$]	0.0014
Left ventricular ejection fraction (%)	69.2 ± 3.6	63.1 ± 7.8	56.8 ± 3.6[#]	0.0205
**Biochemistry Data**				
BUN (mg/dL)	22.4 ± 7.4	28.0 ± 4.5	25.9 ± 1.1	0.3302
Creatinine (mg/dL)	0.11 ± 0.02	0.13 ± 0.01	0.13 ± 0.02	0.1099
ALT (IU/L)	40.8 ± 12.0	50.9 ± 10.7	57.9 ± 18.1	0.2701
Triglyceride (mg/dL)	84.0 ± 11.8	67.1 ± 12.0	82.8 ± 7.3	0.0903
Total cholesterol (mg/dL)	67.6 ± 2.9	71.3 ± 4.8	75.7 ± 3.6[#]	0.0458
LDL-C (mg/dL)	4.6 ± 1.2	4.8 ± 0.4	6.0 ± 0.7	0.0734
HDL-C (mg/dL)	55.0 ± 4.3	63.0 ± 5.3	59.5 ± 1.1	0.0587
VLDL-C (mg/mL)	8.1 ± 3.0	3.5 ± 1.5*	10.3 ± 3.0	0.0134
Glucose (mg/dL)	177.8 ± 27.8	180.3 ± 23.9	191.3 ± 16.9	0.6948

Data are presented as mean ± standard deviation. With Tukey's multiple comparison test: *Comparisons significant for Normal-VLDL versus Control; #Comparison significant for MetS-VLDL versus Control; $Comparison significant for MetS-VLDL versus Normal-VLDL.
